# Diversity and Genetic Variation among *Brevipalpus* Populations from Brazil and Mexico

**DOI:** 10.1371/journal.pone.0133861

**Published:** 2015-07-24

**Authors:** E. J. Sánchez-Velázquez, M. T. Santillán-Galicia, V. M. Novelli, M. A. Nunes, G. Mora-Aguilera, J. M. Valdez-Carrasco, G. Otero-Colina, J. Freitas-Astúa

**Affiliations:** 1 Postgrado en Fitosanidad-Entomología y Acarología. Colegio de Postgraduados, Montecillo, Edo. de Mexico, Mexico; 2 Centro APTA Citros Sylvio Moreira-IAC, Cordeirópolis, Sao Paulo, Brazil; 3 Postgrado en Fitosanidad-Fitopatología. Colegio de Postgraduados, Montecillo, Edo. de Mexico, Mexico; University of Arkansas, UNITED STATES

## Abstract

*Brevipalpus phoenicis* s.l. is an economically important vector of the *Citrus leprosis virus*-C (CiLV-C), one of the most severe diseases attacking citrus orchards worldwide. Effective control strategies for this mite should be designed based on basic information including its population structure, and particularly the factors that influence its dynamics. We sampled sweet orange orchards extensively in eight locations in Brazil and 12 in Mexico. Population genetic structure and genetic variation between both countries, among locations and among sampling sites within locations were evaluated by analysing nucleotide sequence data from fragments of the mitochondrial cytochrome oxidase subunit I (COI). In both countries, *B*. *yothersi* was the most common species and was found in almost all locations. Individuals from *B*. *papayensis* were found in two locations in Brazil. *Brevipalpus yothersi* populations collected in Brazil were more genetically diverse (14 haplotypes) than Mexican populations (four haplotypes). Although geographical origin had a low but significant effect (ca. 25%) on the population structure, the greatest effect was from the within location comparison (37.02 %). Potential factors driving our results were discussed.

## Introduction

Citrus crops are important worldwide and sweet orange, *Citrus sinensis* (L.) Osbeck (Sapindales: Rutaceae), is the most economically important species produced [1]. Currently, Brazil is the world's largest producer of oranges followed by the United States, China, India and Mexico [1]. Currently, one of the most important viral diseases affecting citrus production in Brazil is leprosis, caused by *Citrus leprosis virus* C (CiLV-C) [2]. Transmission of this virus has been related to mite species in the genus *Brevipalpus*, specifically *B*. *phoenicis* (Geijskes) [3–5]. However, the existence of a species complex within *B*. *phoenicis* [6] makes it more difficult to assess the true role of each species in the transmission of CiLV-C. Previously, this species complex has been referred to as *B*. *phoenicis* group species A-G [6], but recently the species status of *B*. *phoenicis* has been revised and the putative species referred to as species groups within *B*. *phoenicis* have been elevated to separate species, specifically: *B*. *azores* Beard & Ochoa, *B*. *feresi* Ochoa & Beard, *B*. *ferraguti* Ochoa & Beard and *B*. *tucuman* Beard & Ochoa. Furthermore, four species previously considered as synonyms of *B*. *phoenicis* have been confirmed as separate species, specifically: *B*. *hondurani* Evans, *B*. *papayensis* Baker, *B*. *phoenicis* s.s. (Geijskes) and *B*. *yothersi* Baker [7]. All the information reported so far regarding *B*. *phoenicis* has been done without considering the existence of a species complex. Therefore, we will consider this species as *B*. *phoenicis sensu lato*, and wherever possible we will refer to the new species description made by Beard et al. [7].

*Brevipalpus phoenicis* s.l. is a tropical and subtropical species that feeds on at least 486 host plants including agricultural, ornamental and weed species [8–10]. Reproduction of *Brevipalpus* mite species is by thelytokous parthenogenesis with females producing females that are genetically similar [11]. Females are haploid with two chromosomes and males are rarely found [12]. Interestingly, asexuality in this species complex is due to the presence of bacteria from the genus *Cardinium* [13]. When these bacteria were removed from mite populations, some males were produced [14], although they appeared to be unable to reproduce [15]. Although some males can be produced, perhaps by an inefficient transmission of the bacteria, as suggested by Groot et al. [13], the lack of functional males induces parthenogenesis as the main mode of reproduction in this species [14]. The fact that this species is polyphagous contradicts the hypothesis that asexual species are unsuccessful colonizers of different environments [16]. However, it has also been reported that successful colonization of new host plants depends on the original host plant. For example, *B*. *phoencisis* s.l originating from acerola (*Malpighia glabra* L. Malpighiales: Malpighiaceae) did not adapt to two new host plants tested—sweet orange and hibiscus (*Hibiscus rosa-sinensis*, L. Malvales: Malvaceae). However, populations originating from sweet orange adapted well to the other two host plant species [17]. It is not known whether these results may be explained by the recent description of cryptic species within *B*. *phoenicis* s.l. [7] where each new species might be associated with different host ranges.

In Brazil, only *B*. *phoenicis* has been reported causing damage on citrus, mainly due to the transmission of CiLV-C [2, 18]. However, in Mexico *B*. *phoenicis* s.l. is part of a community of mites on citrus that also include *Brevipalpus obovatus* (Donnadieu) and *Brevipalpus californicus* (Banks) [19–23]. In both countries, the presence of *B*. *phoenicis* s.l. is consistent with damage, suggesting the effect of CiLV-C in Mexico may become as severe as in Brazil if no new control strategies are designed and implemented. Currently, the most common strategy to control CiLV-C is by controlling mite populations [24–25]. In Brazil, the citrus industry spends $US 62 million per year on the control of *B*. *phoenicis* [26]. Effective control strategies for this mite should be based on an understanding of its population structure, and particularly the factors that influence its dynamics [27]. In Mexico, to our knowledge, there have only been two studies on the population dynamics and distribution of *B*. *phoenicis* on different citrus species and in different regions of Mexico [23–28].

*Brevipalpus yothersi* (formerly *B*. *phoenicis* morphotype B) has been reported in both Brazil and Mexico, but *B*. *papayensis* (formerly *B*. *phoenicis* morphotype C) has only been reported from Brazil [29]. Despite studies determining genetic population structure and genetic variation [30], and the recent publication of Beard et al. [7], studies of different species within the *B*. *phoenicis* s.l. species complex in Brazilian and Mexican populations are practically non-existent and therefore urgently needed. There are several molecular markers used to resolve taxonomic relationships and quantify genetic variation within the same population of a particular mite species [31, 32]. DNA sequence information of the mitochondrial cytochrome oxidase subunit I (COI) has also been used previously to quantify genetic variation and population structure of other mite pests on citrus [33] and to aid taxonomic identification of mites within Tetranychidae and Tenuipalpidae [34]. Improved analyses of genetic diversity and population structure over both large and local geographic scales are important to understand the factors affecting population dynamics and to design effective control strategies [27, 35].

With this aim in mind, we took extensive samples of mites from different locations in Mexico and Brazil and compared the species diversity, genetic diversity and population structure of the *B*. *phoenicis* s.l. species complex using COI sequence information to infer relationships between haplotypes and evaluate genetic differentiation among different populations.

## Material and Methods

### Sampling of mites

*Brevipalpus* mites were collected from sweet orange orchards. In total, we sampled 35 orchards that were distributed as follows: one orchard at each of 11 locations in Brazil (Table 1) from five states, and one orchard at each of 24 locations in Mexico (Table 1) from three states. Collections were made from September to November 2012 in Brazil, and from February to July 2013 in Mexico. At each location in Mexico, samples of leaves, branches and fruits were taken from each of five trees within the orchard (each tree represented one sample) and returned to the laboratory. The trees were selected based on their position in the orchard, one from each of the four corners and one from the centre. In the laboratory the plant material from each tree was searched for mites using a binocular stereomicroscope. Adult mites were collected using a fine brush and deposited into microtubes containing absolute ethanol. Where possible up to 100 individuals were collected from each tree. When only very few mites were found, they were allowed to reproduce on sweet orange fruits under laboratory conditions, following the methods proposed by Chiavegato-Gonzaga [36], to acquire sufficient numbers for identification and analysis. Although five samples were always taken from each location, some had no mites at all and therefore, the final number of trees sampled differed among locations. In Brazil, mite samples were collected and processed in a similar way except from the localities of Gurupi, Sao José Castanhal and Capitao Poco, where mites were stored in 70% ethanol. Before processing, these mites were lyophilized for 20 min to remove the alcohol. The study was conducted in private orchards with the permission of the landowners. The field studies did not involve endangered or protected species. In total 59 samples of mites were collected (Table 1).

**Table 1:** Details of the *Brevipalpus* species sampled in this study. These include those collected from different sites in Brazil and Mexico and also reference DNA material used for genetic comparisons.

**Location (city, state, country)**	**Code**	**Orange variety**	**Haplotype** **‡**	**GenBank accesion number**	**Geographical coordinates**
Pouso Alegre, Minas Gerais, Brazil	POA-1	Pêra	H1	KF954950**	22.266181S, 46.008686W
	POA-2	Pêra	H2	KF954951**	22.266181S, 46.008686W
	POA-3	Pêra	H1	KF954952**	22.266181S, 46.008686W
	POA-4	Pêra	H3	KF954953**	22.266181S, 46.008686W
Lavras, Minas Gerais, Brazil	LAV-1	Pêra	H1	KF954956**	21.287306S, 44.988942W
	LAV-2	Pêra	H2	KF954957**	21.287306S, 44.988942W
São José do Rio Preto, São Paulo, Brazil	SJRPB-1	Bahia	H12	KF954964	20.867119S, 49.357336W
	SJRPB-2	Bahia	H8	KF954965	20.867119S, 49.357336W
	SJRPB-3	Bahia	H13	KF954966	20.867119S, 49.357336W
	SJRPL-1	Lima	H14	KF954967	20.867119S, 49.357336W
	SJRPL-2	Lima	H15	KF954968	20.867119S, 49.357336W
	SJRPL-3	Lima	H16	KF954969	20.867119S, 49.357336W
	SJRPP-3	Pêra	H17	KF954970	20.867119S, 49.357336W
Terenos, Mato Grosso do Sul, Brazil	MS-1	Pêra	H6	KF954958	20.428611S, 55.008889W
Gurupi, Tocantins, Brazil	GUR-1	No commercial	H5	KF954954	11.746844S, 49.049178W
	GUR-3	No commercial	H5	KF954955	11.746844S, 49.049178W
Palmas, Tocantins, Brazil	PAP3-3	No commercial	H7	KF954959	10.291125S, 48.2909W
	PAP4-1	No commercial	H8	KF954960	10.291125S, 48.2909W
	PAP4-3	No commercial	H9	KF954961	10.291125S, 48.2909W
São José Castanhal, Pará, Brazil	SJC-1	Pêra	H10	KF954962	1.43325S, 53.14735W
	SJC-3	Pêra	H11	KF954963	1.43325S, 53.14735W
Capitão Poço, Pará, Brazil	CP2-1	Pêra	H4	KF954971	1.825639S, 53.10225W
Ocozocoautla de Espinosa, Chiapas, Mexico	O1a-1	Valencia	H8	KF954987	16.972417N, 93.503778W
	O1a-2	Valencia	H8	KF954988	16.972417N, 93.503778W
	O2-1	Valencia	H8	KF954989	17.007528N, 93.468111W
	O2-2	Valencia	H8	KF954990	17.007528N, 93.468111W
	O4-2	Valencia	H8	KF954991	17.134611N, 93.294472W
	O5	No commercial	H19	KF954992	17.033861N, 93.544472W
Tecpatán, Chiapas, Mexico	T6-1	Valencia	H8	KF954998	17.217194N, 93.400667W
	T6-2	Valencia	H8	KF954999	17.217194N, 93.400667W
Copainalá, Chiapas, Mexico	C9	Valencia	H8	KF954972	17.033861N, 93.515W
	C10	Valencia	H8	KF954973	17.135583N, 93.293722W
Ángel Albino Corzo, Chiapas, Mexico	JAL15	Valencia	H8	KF954977	15.878583N, 93.729417W
La Concordia, Chiapas, Mexico	LC16-1	Valencia	H8	KF954978	15.888389N, 93.7235W
	LC16-2	Valencia	H8	KF954979	15.888389N, 93.7235W
	LC17-1	Valencia	H8	KF954980	16.097472N, 92.812361W
	LC17-2	Valencia	H8	KF954981	16.097472N, 92.812361W
	LC18-1	Valencia	H8	KF954982	16.097472N, 92.812361W
	LC18-2	Valencia	H8	KF954983	16.097472N, 92.812361W
Villa Corzo, Chiapas, Mexico	VC19	Valencia	H8	KF955002	16.145472N, 93.016222W
	VC20-1	Valencia	H8	KF955003	16.129639N, 93.031139W
	VC20-2	Valencia	H8	KF955004	16.129639N, 93.031139W
	VC21-1	Valencia	H8	KF955005	16.18625N, 93.064111W
	VC21-2	Valencia	H8	KF955006	16.18625N, 93.064111W
Villa Flores, Chiapas, Mexico	VF22-1	Valencia	H20	KF955007	16.268778N, 93.268528W
	VF22-2	Valencia	H8	KF955008	16.268778N, 93.268528W
Salto de Agua, Chiapas, Mexico	SA23-1	Valencia	H8	KF954993	17.321581N, 92.06575W
	SA24-1	Valencia	H8	KF954994	17.338561N, 92.083811W
	SA24-2	Valencia	H8	KF954995	17.338561N, 92.083811W
	SA25-1	Valencia	H8	KF954996	17.33765N, 92.097989W
	SA25-2	Valencia	H8	KF954997	17.33765N, 92.097989W
Uxpanapa, Veracruz, Mexico	UX26-1	Valencia	H8	KF955000	17.225931N, 94.64535W
	UX26-2	Valencia	H8	KF955001	17.225931N, 94.64535W
Las Choapas, Veracruz, Mexico	LCH27-1	Valencia	H18	KF954984	17.599639N, 93.796519W
	LCH27-2	Valencia	H8	KF954985	17.599639N, 93.796519W
	LCH30	Valencia	H8	KF954986	17.555511N, 93.785331W
Cárdenas, Tabasco, Mexico	CAR28-1	Valencia	H8	KF954974	17.967194N, 93.332361W
	CAR28-2	Valencia	H8	KF954975	17.967194N, 93.332361W
Comalcalco, Tabasco, Mexico	COM29	Valencia	H8	KF954976	18.191419N, 93.4054W
**Sequences retrieved from GenBank used for comparison**					
**Species**	**GenBank accession number**			**Reference**	
*Brevipalpus phoenicis* Type 2	KC291373			Navia et al. [30]	
*B*. *phoenicis* Type 2	KC291372			Navia et al. [30]	
*B*. *phoenicis* Type 1	KC291388			Navia et al. [30]	
*B*. *phoenicis* Type 1	KC291389			Navia et al. [30]	
*Brevipalpus obovatus*	KC291383			Navia et al. [30]	
*B*. *obovatus*	KC291384			Navia et al. [30]	
*Brevipalpus chilensis*	KC291398			Navia et al. [30]	
*B*. *chilensis*	KC291399			Navia et al. [30]	
*Brevipalpus californicus*	DQ789591			Groot and Breeuwer [13]	
*B*. *californicus*	KC291402			Navia et al. [30]	
*Cenopalpus pulcher*	AY320029			Rodriguez et al. [49]	

All living samples from Brazil and Mexico were identified morphologically and DNA extracted for sequencing and phylogenetic analysis: Samples from Brazil and Mexico samples were identified as *B*. *yothersi* or *B*. *papayensis* (**),

^‡^ = Haplotypes obtained by a maximum parsimony network using TCS 1.21.

### Morphological identification

Only adult females were used for morphological identification. From each sample, 30 mites were separated and processed for microscopy. Mites were deposited in Hoyer mounting liquid on a glass slide and covered with a coverslip [37]. The glass slides were maintained at 45°C for 15 days. All slides were cleaned using 70% ethanol and cotton swabs. The coverslip was sealed using transparent nail polish. Species identifications were made according to dichotomous keys [19, 20, 22]. Species determination within the *B*. *phoenicis* s.l. species complex was done using the descriptions of Beard et al [7]. All specimens were examined by phase and differential interference contrast (DIC) microscopy.

### Analysis of genetic variation among populations of identified *Brevipalpus* species

DNA was extracted from ten adults per sample using the DNA extraction kit DNeasy Blood & Tissue (Qiagen, Germantown, MD, USA) following the manufacturer’s instructions. Partial sequences of the COI gene were obtained using the primers DNF- 5’ TGA TTT TTT GGT CAC CCA GAA G 3’ and DNR- 5’ TAC AGC TCC TAT AGA TAA AAC 3’ [34]. PCR was performed in a 25 μL reaction volume containing 2.5 μL of buffer 10X (600 mM Tris-SO4 (pH 8.9), 180 mM ammonium sulphate), 1 mM of MgSO_4_, 0.2 μM of each primer, 0.2 mM of dNTP′s, 0.5 μL of Platinum Taq High Fidelity DNA Polymerase (Invitrogen, Life Technologies, Carlsbad, California, USA) and 5 μL (approx. 20 ng) of DNA.

PCR amplifications were performed with an Applied Biosystems thermocycler (Life Technologies Corporation, Foster, CA, USA) in Brazil and a MyCycler (BIO-RAD Laboratories Inc., Hercules, CA, USA) thermocycler in Mexico, using the same thermal conditions: one cycle of 4 min at 94°C, followed by 35 cycles of 60 s at 94°C, 60 s at 54°C and 60 s at 72°C with a final extension at 72°C for 4 min. PCR products were visualised on 1.2% agarose gels in 1X TAE. GelPilot 100 bp Plus (QIAGEN, GmbH, Hilden, Germany) size markers were used. The gels were stained with ethidium bromide (0.1μg/mL) and photographed.

Sequencing of the Brazilian samples was done at the Centro APTA Citros ‘Sylvio Moreira’ IAC, Brazil. Sequencing reactions were performed using the BigDye Terminator 3.1 kit (Perkin Elmer, Foster City, CA) following the manufacturer’s instructions. Mexican samples were sent to Macrogen Inc. (South Korea) for direct sequencing.

### Data analysis

Sequence traces were assembled using BioEdit [38]. For this, clear and unambiguous peaks representing each base were located in each raw sequence trace. Data beyond this point at both ends of the sequence were discarded. The same was done for forward and reverse sequences for each sample and, by combining both sequences (forward and reverse) we were able to generate only one sequence for each sample. All sequences were truncated to the same length (352 bp) to eliminate missing data. Multiple sequence alignments were made using Clustal W [39]. Maximum parsimony, maximum likelihood and neighbour joining analyses were done using Molecular Evolutionary Genetic Analysis (MEGA) software ver. 5.0 for Windows [40], using the Close-Neighbour-Interchange algorithm. The robustness of branches was estimated by bootstrap analysis with 1000 repeated samplings of the data [41]. Tree reconstructions were made excluding non-synonymous substitutions, without any effect on tree topology. We show the tree including all sites. Sequences from related species within the genus *Brevipalpus* were retrieved from GenBank and used for comparison (Table 1). A *Cenopalpus pulcher* (Canestrini and Fanzago) (Acari: Tenuipalpidae) sequence was used as the outgroup for this analysis. In addition, the Nei-Gojobori method [42], as implemented in the Z test in the program MEGA 5.0 [40], was used to compute the synonymous and nonsynonymous distances at a 5% significance level. Genetic differences among haplotypes were represented in a maximum parsimony network [43] using TCS 1.21 [44] with 95% confidence in the connection limit (limits of parsimony) and where gaps were treated as a 5th state. Haplotype and nucleotide diversity [45] were calculated using DnaSP v5 [46]. Finally, the partition of genetic variation between countries, among populations (locations) within each country and amongst all populations regardless of country or location of origin was estimated only for *B*. *yothersi* populations using analysis of molecular variance (AMOVA) and by computing F-statistics using Arlequin v. 3.5 [47] with 10000 permutations. No AMOVA analysis was attempted for *B*. *papayensis* as there were only sufficient samples from Brazil for molecular study.

## Results

All specimens were identified morphologically from Brazilian and Mexican samples, and the majority were from *Brevipalpus yothersi* and *B*. *papayensis*. In only three samples (O2, O4 and O5, Table 1), *B*. *californicus* was also found. When two species were found, single adults were placed on oranges (one mite per orange and maintained separately to avoid any cross-contamination), and allowed to reproduce parthenogenetically. When a significant number of mites were produced (more than 100), ca. five mites per orange were mounted, identified, and only the sample oranges containing *B*. *yothersi* or *B*. *papayensis* were used in the genetic analysis. Those sample oranges with *B*. *californicus* were discarded because they were in such low numbers that any meaningful molecular analysis of *B*. *californicus* was not possible.

**Fig 1:**
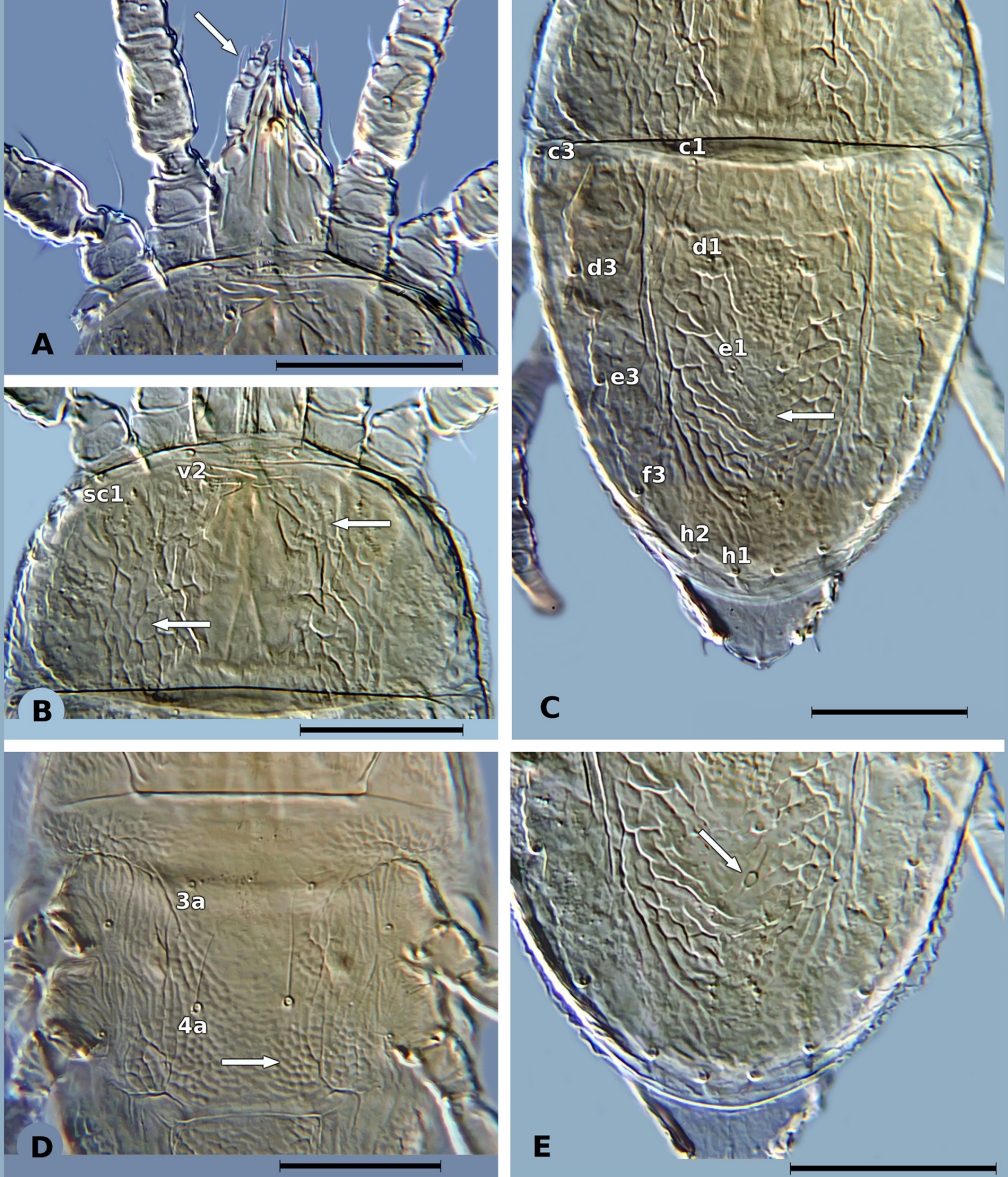
Morphological characteristics of *B*. *yothersi*. A) Palp femur with barbed, setiform dorsal seta. B) Cuticle of the propodosoma, sc1 = scapular seta, v2 = vertical seta, white arrows show anterior and posterior reticulation. C) Dorsal cuticle of the opisthosoma, dorsal opisthosomal setae: c1, c3, d1, d3, e1, e3, f3, h1, h2; white arrow shows ‘V’ shaped reticulated area. D) Ventral view of the cuticle between aggential setae 3a and 4a, white arrow shows rounded reticulations. E) White arrow shows spermatheca. Black line represents 50 μM.

**Fig 2:**
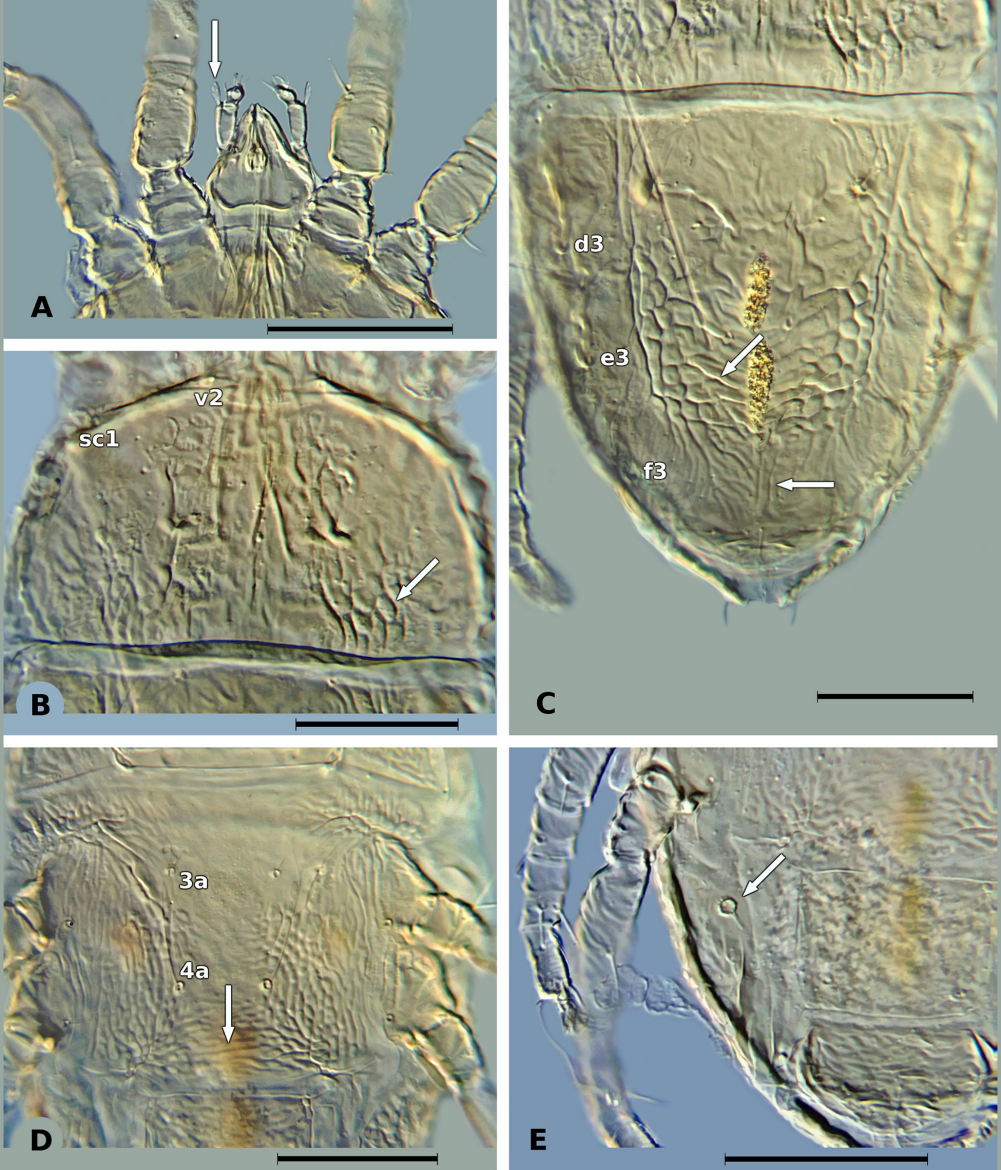
Morphological characteristics of *B*. *papayensis*. A) Palp femur with barbed, setiform dorsal seta. B) Cuticle of the propodosoma, sc1 = scapular seta, v2 = vertical seta, white arrow shows anterior and posterior reticulation. C) Dorsal cuticle of the opisthosoma, dorsal opisthosomal setae: c1, c3, d1, d3, e1, e3, f3, h1, h2; white arrow shows reticulation between setae e1 and h1 starting as transverse folds and becoming longitudinal towards h1. D) Ventral view of the cuticle between aggential setae 3a and 4a, white arrow shows reticulations forming transverse bands. E) White arrow shows spermatheca. The black line represents 50 μM.

In both countries, *B*. *yothersi* (Fig 1) was the most common species and was found in almost all locations. Individuals from *B*. *papayensis* (Fig 2) were found in two locations in Brazil (Pouse Alegre and Lavras, Minas Gerais, Table 1), and in only one location in Mexico (O1: Ocozocoautla de Espinosa) (Table 1), where *B*. *yothersi* was also found. As very low numbers of mites (less than ten mites) from both *B*. *yothersi* and *B*. *papayensis* were found in location O1, increasing their population by rearing them on sweet oranges was attempted in the laboratory. However, successful reproduction was only achieved for *B*. *yothersi* meaning there were insufficient Mexican *B*. *papayensis* for genetic analysis. The morphological characteristics used to separate *B*. *yothersi* and *B*. *papayensis*, are listed in Table 2, and illustrated in Figs 1 and 2.

**Table 2:** Morphological characteristics used to separate *B*. *yothersi and B*. *papayensis* (Beard et al. [7]).

Morphological characteristic	*B*. *yothersi*	*B*. *papayensis*
Dorsal palp femur seta	Setiform and barbed (Fig 1A)	Broadly setiform and barbed (Fig 2A)
Sublateral region of propodosoma	Posterior region forming large cells, anterior region minus reticulate (Fig 1B)	Reticulations like large cells only in the posterior end (Fig 2B)
Opisthosoma	Reticulation between setae e1 and h1 with “V” shaped folds (Fig 1C)	Reticulation between setae e1 and h1 starting with transverse folds abruptly becoming longitudinal folds towards h1 (Fig 2C)
Ventral region posterior to setae 4a	Rounded reticulations (Fig 1D)	Elongate reticulations forming transverse bands (Fig 2D)
Spermatheca	With a long narrow duct, which merges to an oval vesicle with small distal stipe (Fig 1E)	With a long moderately thick duct, which ends in a spherical vesicle with a crown of small projections (Fig 2E)

### Genetic variation among *B*. *yothersi* and *B*. *papayensis* populations

**Fig 3:**
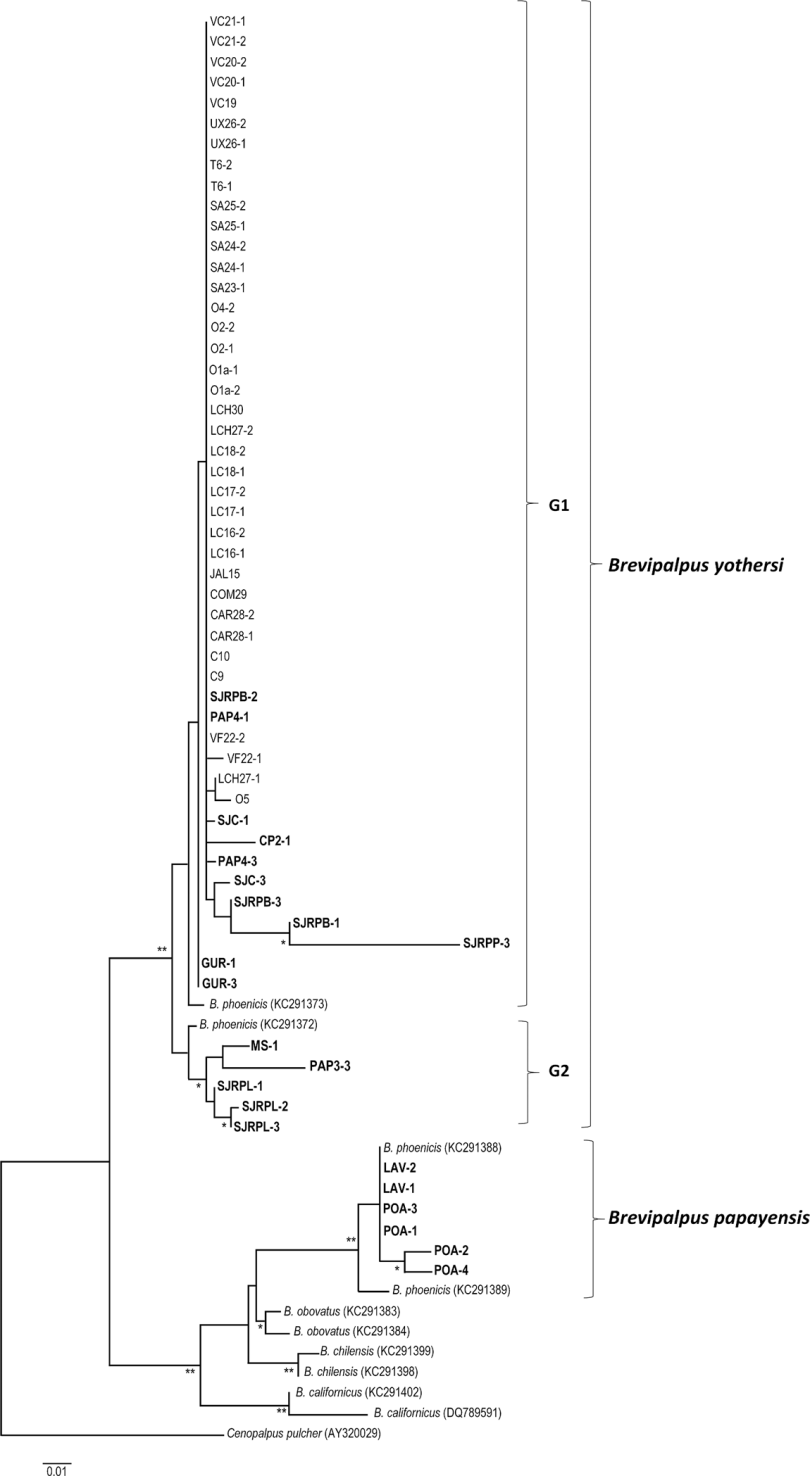
Dendrogram inferred from maximum likelihood, maximum parsimony and neighbour joining analyses of COI data from *B*. *yothersi* and *B*. *papayensis*. Samples in bold were collected in Brazil. Other *Brevipalpus* species used as reference species and *Cenopalpus pulcher* (Canestrini and Fanzago) (Acari: Tenuipalpidae) used as the outgroup, are labelled according to their GenBank accession numbers. Only bootstrap values above 90% with the three analyses were considered. Significance of values obtained with the three analyses are represented by asterisks (* ≥ 90%, ** ≥ 95%). G1 = group 1, G2 = group 2. Scale bar represents the number of nucleotide substitutions after maximum likelihood analysis.

Fifty-nine partial COI sequences were obtained which, after alignment and trimming, resulted in a final sequence length of 352 bp. The number of non-synonymous substitutions was greater than the number of synonymous substitutions per site (Z = 2.271, P = 0.012). These sequences contained 266 non-variable sites, 86 variable sites and 48 parsimony-informative sites. GenBank accession numbers are shown in Table 1. Phylogenetic analyses showed a clear separation among species within the genus *Brevipalpus* with all bootstrap values above 90% (Fig 3). All samples morphologically identified as *B*. *yothersi* were grouped together with the *B*. *phoenicis* type 2 [32] (now considered as *B*. *yothersi*) sequences retrieved from GenBank that were used as a reference. Samples morphologically identified as *B*. *papayensis* were grouped together with the *B*. *phoenicis* type 1 [32] (now considered as *B*. *papayensis*) sequences (Fig 3). All samples of *B*. *papayensis* evaluated were from Brazil only, while the samples of *B*. *yothersi* evaluated were collected from both Brazil and Mexico. The *B*. *yothersi* samples could be further separated into two distinct groups, G1 and G2 (Fig 3). Group G1 contained 48 samples, with samples SJRPB-1 and SJRPP-3 forming a distinct group separated from the other samples with a bootstrap value above 90%. Group G2 contained five samples, all from Brazil, with samples SJRPL-2 and SJRPL-3 forming a distinct group, followed by sample SJRPL-1 with bootstrap values above 90% (Fig 3).

**Fig 4:**
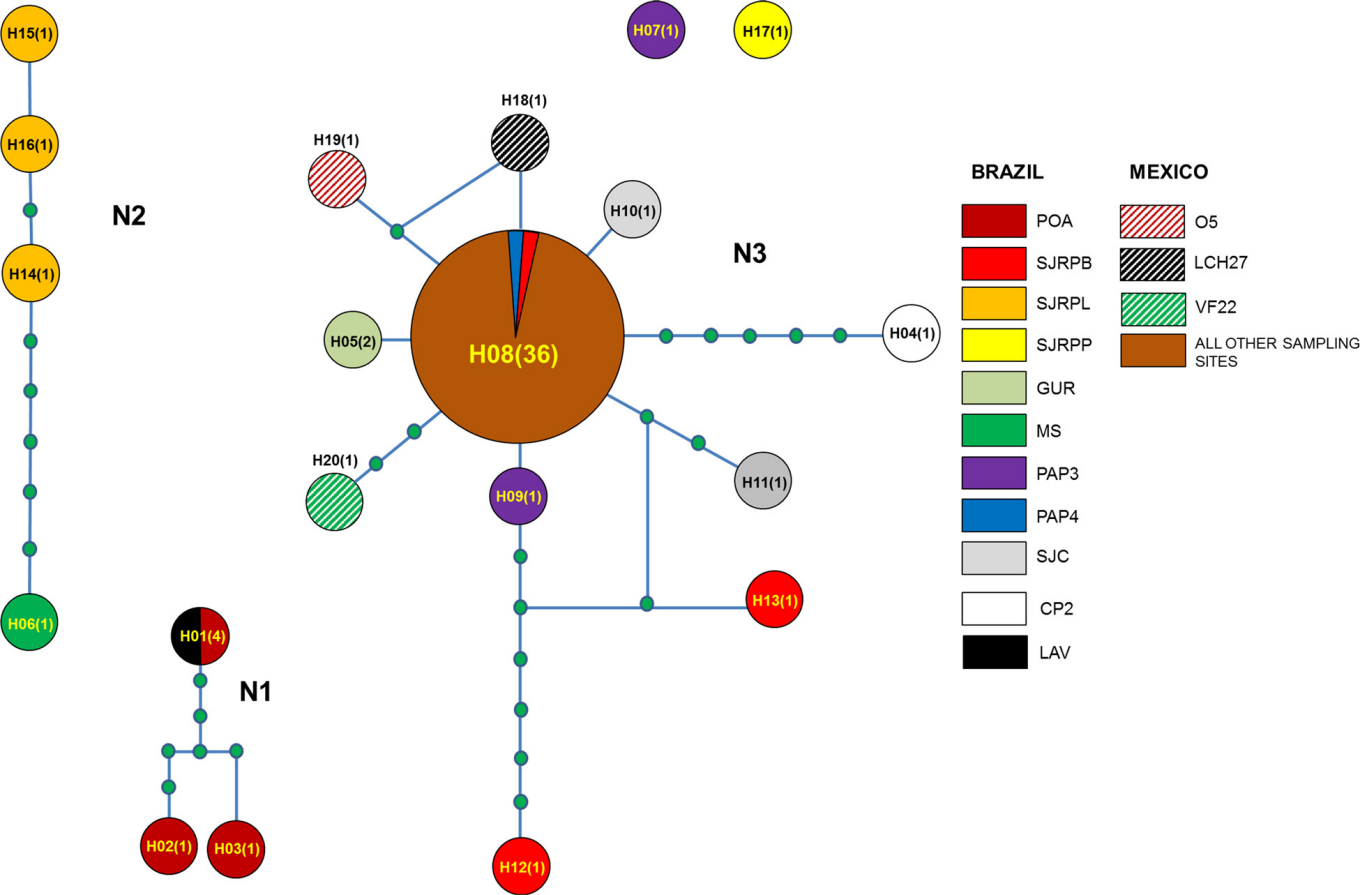
The most parsimonious haplotype network for the 20 haplotypes found in *B*. *yothersi and B*. *papayensis*. Colours indicate different sampling locations where each haplotype is present in Brazil and Mexico (Table 1). Haplotypes are connected with a 95% confidence limit. Each line in the network represents a single mutational change. Small circles indicate missing haplotypes. Numbers of samples per haplotype are shown in parentheses. N1-3 = network 1–3.

Haplotype Network analysis showed the existence of 20 haplotypes from 35 sampled trees at 20 orchard localities in Mexico and Brazil (Table 1; Fig 4). There were three discreet networks, network N1 contained only samples identified as *B*. *papayensis*, and networks N2 and N3 contained only samples identified as *B*. *yothersi*. Network N1 contained three haplotypes: H01, H02 and H03. H02 and H03 were each found in only one sample but H01 was found in four samples; all six samples were collected in Brazil. The second network (N2) contained four haplotypes, each found in only one sample and all from Brazil. The third and largest network (N3) contained 11 haplotypes that were found in 47 samples. The most common haplotype was H08, which was found in 36 samples, all from Mexico except PAP4-1 and SJRPB-2, which were from Brazil. Finally, there were two independent haplotypes from Brazil that did not belong to any network (H07 and H17) and were each found in only one sample (Fig 4). There was greater haplotype (0.966±0.028) and nucleotide (0.06248±0.00710) diversity in *B*. *yothersi* populations from Brazil, where 17 haplotypes were recorded from 22 samples analysed, compared with the haplotype (0.158±0.080) and nucleotide (0.000092±0.00152) diversity found in Mexico with only four haplotypes from 37 samples analysed.

AMOVA analysis (Table 3) revealed that the greatest amount of variation among *B*. *yothersi* populations could be accounted for by differences between individuals within each population (81.15%), followed by differences between Brazil and Mexico (16.33%) and finally by differences among populations within each country (2.52%). Although the figure for the difference between Brazil and Mexico was not the greatest, it showed a highly significant P value (P<0.0001) suggesting a geographically structured population (Table 3).

**Table 3:** Results of AMOVA analysis of COI sequences from *B*. *yothersi* populations.

Source of variation	d.f.	Sum of squares	Variance components	% of variation explained
Between groups (Brazil and Mexico)	1	30.384	0.84	16.33***
Among populations within groups	16	73.874	0.12	2.52**
Within populations	46	192.024	4.17	81.15**

**P<0.04,

***P<0.0001

## Discussion

Combining morphological and genetic analyses is a powerful way to obtain the maximum information on taxonomic and genetic variation in any species [48]. Considerable taxonomic and genetic information is available for *B*. *phoenicis* s.l. [29, 30, 49–51], but there remain many gaps in understanding genetic variation within and between populations and species. For example, the recent report raising many morphotypes within the *B*. *phoenicis* species complex to species level [7], and the fact that specific relationships between virus and host have been reported [52], suggests the necessity for assessing the ability of each of these new species to transmit CiLV-C. Currently, only the relationship between CiLV-C and *B*. *yothersi* has been reported [53], so the relationship between CiLV-C and *B*. *papayensis* remains to be investigated. In addition, the factors that drive genetic variation within these new species, and how this might vary between their geographical origins are poorly understood.

Morphological identification showed the existence of two main species, *B*. *yothersi* and *B*. *papayensis*. Mites from *B*. *papayensis* were only found in Lavras and Pouso Alegre (Minas Gerais), Brazil and Ocozocoautla de Espinosa (Chiapas), Mexico, the latter representing the first report of this species from Mexico. Unfortunately, there were insufficient numbers of mites for DNA extraction to evaluate genetic variation as was done with the samples from Brazil. *Brevipalpus papayensis* specimens in this study were all collected from citrus orchards located in coffee growing regions, which was consistent with previous studies in Brazil [30]. More sampling near coffee plantations must be done to confirm this association.

The use of COI sequencing has been used previously to study *B*. *phoenicis* s.l. populations from Brazil, Chile, The Netherlands and USA [52]. Using the same marker, Groot and Breeuwer [13] found conflicts between genetic analysis and morphological identification. Navia et al. [30] suggested that these conflicts were due to the existence of cryptic species within the genus *Brevipalpus*, which was later confirmed by Beard et al. [7]. Our study confirms the existence of a species complex within *B*. *phoenicis* s.l. (Figs 1 and 2), which corresponds to the species *B*. *yothersi* and *B*. *papayensis* reported by Beard et al. [7]. Our data showed that populations from both species were indeed genetically different (Figs 3 and 4), which was clearly demonstrated in the Brazilian populations. Unfortunately, insufficient individuals from *B*. *papayensis* were collected in Mexico to allow us to confirm this genetic separation in Mexican populations. Although Beard et al. [7] have raised all of these morphotypes to species level, we still suggest that their study should be complemented with molecular data from more than one gene [54].

The haplotype network (Fig 4) showed the existence of three discrete networks where network 1 (Fig 4) contained only samples from *B*. *papayensis*. The existence of the other networks (2 and 3) including haplotypes 7 and 17 can be considered as a consequence of the genetic variation within *B*. *yothersi*. The majority of the specimens collected in both countries, corresponded to *B*. *yothersi*, and within this species, greater genetic variation was observed in populations from Brazil than in populations from Mexico (Fig 4). In fact, the majority of the Mexican populations were clustered in one group following phylogenetic analyses (Fig 3) and there were only four haplotypes (Fig 4). Although, thelytokous parthenogenesis may be responsible, it is also likely that selection pressures on *B*. *yothersi* populations, such as the number and type of acaricide applications made, are greater in Brazil compared to Mexico, resulting in genetically more diverse populations in Brazil, This mechanism has also been suggested to account for variability in genetic diversity in *Panonychus citri* (Acari: Tetranychidae), another important mite pest in citrus orchards worldwide. ITS1 sequence analysis revealed greatest genetic diversity among *P*. *citri* populations from different locations in China, where the control of this mite relied most heavily on acaricides [55].

Additionally, it is possible that the host plant may also be playing a significant role in the greater genetic diversity found in the Brazilian samples of *B*. *yothersi*. Although all Brazilian mites were collected from sweet orange (*C*. *sinensis*) orchards, samples were collected from four different varieties, Pêra, Lima, Bahia and a non-commercial variety. With the exception of one sample (O5) all samples from Mexico were from the same variety, Valencia (Table 1). This may contribute to a lack of genetic diversity within *B*. *yothersi* populations in Mexico. Host-association differentiation (HAD) has been described for mites previously. For example the existence of a large number of genetically distinct lineages of the mite *Aceria tosichella* Keifer (Prostigmata: Eriophyidae) were associated with specific plant hosts, regardless of geographic origin [56]. Based on this, we propose that sampling different varieties of *C*. *sinensis* may have contributed to the existence of more haplotypes within *B*. *yothersi* populations in Brazil compared with Mexico where only one orange variety is normally grown by producers. Although they reproduce clonally, genetically distinct lineages or haplotypes of *B*. *yothersi* may be related to the orange variety on which they were collected suggesting some level of host-plant specialisation, a process described as the Frozen Niche Variation model (FNV) as previously reported for *B*. *phoenicis* s.l. by Groot et al. [17]. Currently, we are performing more studies to assess the role of the host plant in shaping patterns of genetic variation in *B*. *yothersi* and *B*. *papayensis* populations.

In summary, morphological and genetic analysis has demonstrated the existence of *B*. *yothersi* and *B*. *papayensis* populations collected in Brazil and Mexico, although genetic variation between these two species was only confirmed for Brazilian populations. In both countries, *B*. *yothersi* was the most abundant species and was more genetically diverse in Brazil than in Mexico. The existence of these two species in Mexico and Brazil requires research, including behavioural and ecological studies, as it is likely that one species may be more efficient in transmitting CiLV-V. Such information should be included in the design of monitoring and control programs, especially for *B*. *yothersi*, which was the most abundant species compared to *B*. *papayensis*.
